# Passing sutures

**Published:** 2023-12-01

**Authors:** Jacqueline Newton, Rebecca Jones

**Affiliations:** 1 Staff Nurse: Flying Eye Hospital, Orbis International, Cape Town, South Africa.; 2 Ophthalmology Registrar: Cheltenham General Hospital, Cheltenham, UK.

**It is important to load sutures correctly before passing them to the surgeon. Pay attention to where and how you grip the needle, and be aware that a different position may be needed for left-handed surgeons.**

Most sutures used in ophthalmic surgery are loaded onto curved needles, which should always be passed with the sharp tip and swage curving up, towards the ceiling.

Ask the surgeon ahead of time how they would like their needle to be loaded (or held) in the needle holder. Also, check whether they are right-handed or left-handed.

## Forehand or backhand pass?

Most surgeons prefer to make **forehand** suture passes, working from the side of their dominant hand **towards the centre** of the operating field. For a right-handed surgeon, the needle tip must point to the left, and for a left-handed surgeon, it must point to the right.

Ask the surgeon to tell you if they want to make a **backhand** suture pass, i.e., working **away from the centre** of the operating field. A backhand pass for a right-handed surgeon would be loaded in the same orientation as a forehand pass for a left-handed surgeon, and vice versa.

## Loading the needle holder

You will need:

- A needle holder (also known as a needle driver)
- The suture needle, with a suture attached to it
- A second instrument, such as tying forceps

## Steps

**Figure 1:**
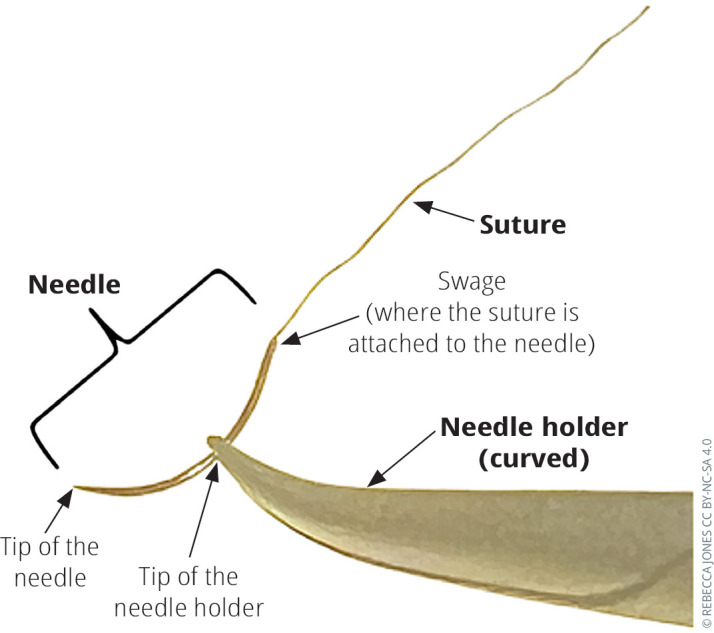
The suture needle, suture, and needle holder. The centre or body of the needle is gripped by the tip of the needle holder. The surgeon is right-handed, so the sharp point of the needle faces to the left.

**Figure 2:**
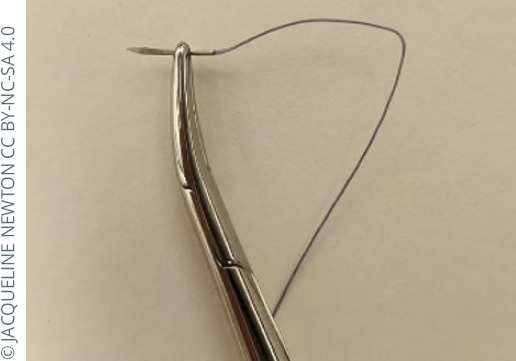
Suture needle gripped correctly for a **forehand pass** by **right-handed** surgeon.

**Figure 3:**
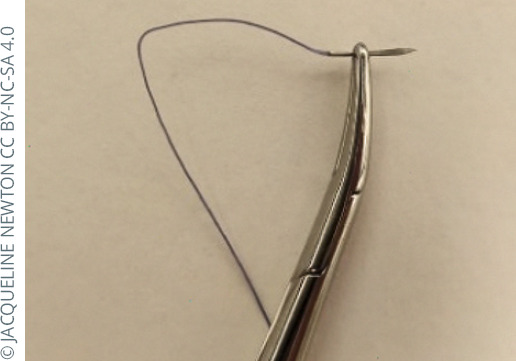
Suture needle gripped correctly for a **forehand pass** by **left-handed** surgeon.

1. Use the second instrument, such as tying forceps, to pick up the needle. Note: **Do not touch the needle with your hands**, even when wearing gloves. This will help to avoid injury.
2. Open the needle holder. Use the **tip** of the needle holder to grip the needle **just to the rear of the centre of the needle**; in other words, slightly closer to the swage than to the tip of the needle. Figures 1 and 2 show the correct position when loading a suture needle for a right-handed surgeon making a forehand pass; Figure 3 is the position for a left-handed surgeon.
3. Grasp the needle holder in the centre and pass to the surgeon.

## Using a curved needle holder

Curved needle holders have a tip that is curved (or bent) to one side; this allows the surgeon more room to manipulate the suturing needle, without the needle holder getting in the way. To grip a suturing needle using a curved needle holder, rotate the needle holder so that the tip bends, or curves, in the direction of the swage (see Figures 2 and 3).

**Tips**

- Avoid gripping the needle close to its point, otherwise the surgeon will not be able to insert the needle deeply enough into the tissue. You may also blunt the sharp needle tip.
- Avoid gripping the needle close to the swage, because you may damage the needle and dislodge the suture.

**This YouTube video has useful practical tips:** bit.ly/CEHJ-suture

